# Comparison of PCR-based detection of *Plasmodium falciparum *infections based on single and multicopy genes

**DOI:** 10.1186/1475-2875-6-112

**Published:** 2007-08-16

**Authors:** Segun I Oyedeji, Henrietta O Awobode, Gamaliel C Monday, Eric Kendjo, Peter G Kremsner, Jürgen F Kun

**Affiliations:** 1 Institute for Tropical Medicine, Department of Parasitology, University of Tübingen Wilhelmstrasse 27, D-72074, Tübingen, Germany; 2 Parasitology Unit, Department of Zoology, University of Ibadan, Ibadan, Nigeria, Africa; 3 Clinical Service Unit, Nasarawa State Ministry of Health, Lafia, Nigeria, Africa; 4 Medical Research Unit, Albert Schweitzer Hospital, Lambaréné, Gabon, Africa

## Abstract

PCR-based assays are the most sensitive and specific methods to detect malaria parasites.

This study compared the diagnostic accuracy of three PCR-based assays that do not only differ in their sequence target, but also in the number of copies of their target region, for the detection of *Plasmodium falciparum *in 401 individuals living in a malaria-endemic area in Nigeria. Compared to a composite reference generated from results of all the 3 PCR assays, the *stevor *gene amplification had a sensitivity of 100% (*Kappa *= 1; 95% CI = 1.000–1.000), 83% (*Kappa *= 0.718; 95% CI = 0.648–0.788) by *SSUrRNA *gene PCR and 71% (*Kappa *= 0.552; 95% CI = 0.478–0.627) by the *msa*-*2 *gene amplification.

Results from this study indicate that the *stevor *gene amplification is the most sensitive technique for the detection of *P. falciparum*. This assay may be an important reference standard, especially when a confirmatory technique with high sensitivity and specificity is needed for ruling out *P. falciparum *infection.

## Background

Prompt and accurate diagnosis is an important tool in the effective management and control of malaria, a disease which accounts for more than a million deaths annually [1,2]. Microscopy remains the cheapest and most commonly used method for malaria diagnosis and relies on the microscopic examination of stained blood films. Microscopy, however, has its own limitations, even when performed by an expert. It is time-consuming and its sensitivity is limited, particularly when parasitaemia is low [3]. Sensitivity and specificity are of crucial importance in any identification method as a false negative result could result in the non-treatment of a potentially fatal disease and conversely a false positive result will expose individuals to unnecessary drug intake, with its associated side-effects and high cost, while leaving the true cause of the illness untreated. Misdiagnosis of malaria, however, could have fatal consequences [4]. It is, therefore, important that a reliable, rapid and efficient method for diagnosis, surveillance and epidemiological studies is identified.

Several new diagnostic techniques have been developed during the last decade, including antigen detection and fluorescence-based assays, which have the added advantage of moving the diagnosis of malaria nearer to the patient and even faster [5,6]. These, also have drawbacks as their sensitivity decreases especially with low parasitaemia or they may have variability in performance: misdiagnosis with such methods have been reported [7-11].

PCR-based assays have been used in diagnosis and as reference or standard for the assessment of the sensitivity and specificity of microscopy and commonly deployed rapid diagnostic tests and may indeed, be of clinical value in some selected situations [12-17]. Examples include: the PCR of *stevor *gene, a multiple copy sequence of the subtelomeric variable open reading frame of *Plasmodium falciparum *which were used in detection or measurement of very low parasitaemia [18,19]; the genus and species-specific PCR, which was designed to amplify portions of the sequences coding for the small subunit ribosomal RNA (*SSUrRNA*) and has the added advantage of being able to detect all the four species of human *Plasmodium*, as well as the quantification of *P. falciparum *[20,21]; and, the PCR typing of highly polymorphic merozoite surface antigens of *P. falciparum*, which has been particularly useful in determining genetic variability of parasite isolates [22] and in detection of microscopically undetected parasites in drug trials [23].

Despite the fact that PCR-based assays are meant to be highly sensitive, it is not known whether this sensitivity is equally good with all amplification techniques in use and high enough to justify their use as reference or standard in the diagnosis of *P. falciparum *infection. In this study, the sensitivity and specificity of three different PCR assays with target to mono- or multi-copy gene for the detection of *P. falciparum *were assessed.

## Methods

### Study site and sample collection

This study was conducted in Lafia, located within the Guinea savanna ecological zone in north-central Nigeria. Within this region, malaria transmission has formerly been described as stable and uniformly intense through most of the year [24,25]. 401 children between 6 months and 8 years of age, from amongst those who reported for routine medical examination on complaint of fever, were enrolled into the study at the Dalhatu Araf Specialist Hospital (DASH), Lafia between November, 2005 and July, 2006. Informed consent was obtained from the parent or guardian of each child prior to being included in the study. Ethical approval for the study was granted by the Ethical Review Committees of the Nasarawa State Ministry of Health and the Dalhatu Araf Specialist Hospital, Lafia. Inclusion criteria are febrile conditions suggestive of malaria which includes chills, history of fever within the preceding 48 h or pyrexia at presentation (axillary temperature >37.5°C), or fever of unknown aetiology. Children with signs or symptoms of severe illness were excluded from the study.

Finger-prick blood samples were collected for thick and thin blood smear and on filter paper for PCR. Two drops of blood were spotted on each filter paper, air dried and individually sealed in plastic bags and stored at room temperature until DNA extraction. Giemsa-stained thick and thin blood films were examined for malaria parasites. Parasitaemia were quantified relative to 250 white blood cells (WBC) on thick films and estimated as parasites per μl assuming a mean WBC of 8,000 per μl of blood. Blood smears were labeled negative if no parasites were seen after examination of 200 oil immersion fields (×1,000) on a thick blood film.

### DNA extraction and PCR

DNA was extracted from the dried blood spots on filter paper using the QIAamp^® ^DNA Mini Kit (Qiagen, Hilden, Germany) following the manufacturer's protocol, and stored at -20°C until further analysis. 150 μl of distilled water was used to elute DNA. All PCR assays include a primary and nested reaction to enhance specificity. Amplification was performed using a BIOMETRA TB1 thermal cycler (Biotron, Göttingen Germany).

#### *SSUrRNA *gene PCR

In all reaction, 2.0 μl of DNA extract were amplified in a final volume of 25 μl containing 2.5 μl ×10 reaction buffer, 100 μM of each dNTPs (dATP, dGTP, dTTP, and dCTP), 0.5 pM of each primer (rPLU5/rPLU6 for the primary reaction and rFAL1/rFAL2 for the nested reaction) and 0.75 units of *Taq *DNA polymerase (Qiagen, Hilden, Germany). Primer sequences (Table 1) are based on the *SSUrRNA *sequences described elsewhere [26]. The PCR programme was: denaturation at 95°C for 5 min followed by 25 cycles (30 cycles in nested) of 1 min at 94°C, 2 min at 60°C and 2 min at 72°C and a final extension period of 5 min at 72°C.

**Table 1:** Nucleotide sequence of primers used in this study

Primer Name	Nucleotide sequence
rPLU5	5'-CCT GTT GTT GCC TTA AAC TTC-3'
rPLU6	5'-TTA AAA TTG TTG CAG TTA AAA CG-3'
rFAL 1	5'-TTA AAC TGG TTT GGG AAA ACC AAA TAT ATT-3'
rFAL 2	5'-ACA CAA TGA ACT CAA TCA TGA CTA CCC GTC-3'
	
P5	5'-GGG AAT TCT TTA TTT GAT GAA GAT G-3'
P18	5'-TTT CA(C/T) CAC CAA ACA TTT CTT-3'
P19	5'-AAT CCA CAT TAT CAC AAT GA-3'
P20	5'-CCG ATT TTA ACA TAA TAT GA-3'
P17	5'-ACA TTA TCA TAA TGA (C/T) CC AGA ACT-3'
P24	5'-GTT TGC AAT AAT TCT TTT TCT AGC-3'
	
MSA 2-1	5'-ATG AAG GTA ATT AAA ACA TTG TCT ATT ATA-3'
MSA 2-4	5'-TTA TAT GAA TAT GGC AAA AGA TAA AAC AAG-3'
MSA 2-2	5'-ACA TTC ATA AAC AAT GCT TAT AAT ATG AGT-3'
MSA 2-3	5'-GAT TAT TTC TAG AAC CAT GCA TAT GTC CAT-3'
FC 27-1	5'-GCA AAT GAA GGT TCT AAT ACT AAT AG-3'
FC 27-2	5'-GCT TTG GGT CCT TCT TCA GTT GAT TC-3'
3D7-1	5'-GCA GAA AGT AAG CCT TCT ACT GGT GCT-3'
3D7-2	5'-GAT TTG TTT CGG CAT TAT TAT GA-3'

#### *stevor *gene PCR

Primary amplification was performed with reaction mixture of 50 μl containing 5.0 μl ×10 reaction buffer, 200 μM of each dNTPs (dATP, dGTP, dTTP, and dCTP), 1.25 units of *Taq *DNA polymerase, 0.4 pM of each primer (P5, P18, P19 and P20) and 5.0 μl of DNA extract. The PCR programme was: denaturation at 93°C for 3 min followed by 25 cycles of 30 sec at 93°C, 50 sec at 50°C and 30 sec at 72°C and a final extension period of 3 min at 72°C. 2.0 μl of the first-round PCR product was used in the second round amplification which was performed with a reaction mixture of 50 μl containing 5.0 μl ×10 reaction buffer, 200 μM of each dNTPs, 1.25 units of *Taq *DNA polymerase and 0.4 pM of each primer (P17 and P24). The primer sequences for the *stevor *PCR are shown in Table 1. PCR conditions for the nested reaction was: denaturation at 93°C for 3 sec followed by 25 cycles of 30 sec at 93°C, 50 sec at 55°C and 30 sec at 72°C and a final extension period of 3 min at 72°C.

#### *msa-2 *gene PCR

The primary reaction was designed to amplify the entire coding region of the *msa-2 *gene using the *msa-2*-1 and *msa-2*-4 primers pairs (Table 1). The reaction mixture was performed in a final volume of 50 μl containing 5.0 μl of DNA extract, 5.0 μl ×10 reaction buffer, 200 μM of each dNTPs (dATP, dGTP, dTTP, and dCTP), 1.25 units of *Taq *DNA polymerase and 0.5 pM of each primer. The PCR programme was: denaturation at 94°C for 5 min followed by 35 cycles of 10 sec at 94°C, 30 sec at 57°C and 40 sec at 72°C and a final extension period of 3 min at 72°C. This was followed by two sets of nested reactions using allelic family-specific primers (FC27 and 3D7). A third reaction was performed to amplify the entire central variable region with the primer pairs *msa-2*-2 and *msa-2*-3 (Table 1), in order to detect sequences that may not be allelic family-specific. All nested reactions were performed in a final volume of 50 μl containing 2.0 μl of PCR product from primary reaction, 5.0 μl ×10 reaction buffer, 200 μM of each dNTPs, 0.5 pM of each primer and 1.25 units of *Taq *DNA polymerase. The PCR programme was: denaturation at 94°C for 5 sec followed by 30 cycles of 10 sec at 94°C, 30 sec at 57°C and 40 sec at 72°C and a final extension period of 3 min at 72°C.

PCR products were subjected to electrophoresis on 1.5–2% agarose gels and visualized by transillumination with ultraviolet light after staining with SYBR^® ^Green. Fragment sizes were calculated relative to the standard size marker (100 bp DNA ladder) using the BioDocAnalyze (Biometra, Göttingen, Germany) computer software package.

All reactions were run in parallel with DNA from negative controls (non-exposed European samples) confirmed negative by the three PCR assays, and DNA from FCR-3 and 3D7 strains as positive controls. This strategy was used to enhance specificity and to ensure that the reaction does not spuriously amplify homologous sequences. For a reaction to remain valid, all negative controls in a run must remain negative while the positive controls must remain positive.

### Statistical analysis

Data were entered into SPSS for Windows software package, version 11.0.0 (SPSS Inc., 2001). Sensitivity, specificity and Cohen's kappa coefficient (κ) with their 95% confidence intervals (CI) were determined using Stata version 9.2 (StataCorp, College Station, Texas). Cohen's kappa coefficient is a measure of the agreement between two tests beyond that expected by chance, where 0 is chance agreement and 1 is perfect agreement [27].

## Results

Among the 401 children enrolled in this study, 169 presented with microscopically confirmed *P. falciparum*. The mean (± SD) age and weight of the children were 40.4 (± 19.2) months and 13.6 (± 3.1) Kg respectively. The baseline characteristics of the study children are shown in Table 2. A composite reference was generated and used as the gold standard to assess the sensitivity of each PCR assay. This was defined as a positive test by at least one of the three PCR assays relative to the positive and negative controls. This method was adopted in order to avoid errors in measuring the sensitivity of the techniques if a particular assay was chosen as the "gold standard" when it does not have 100% sensitivity. Sensitivity was defined as the probability that a truly infected individual will test positive and was assessed relative to the FCR-3 and 3D7 strains as positive controls. Specificity was defined as the probability that a truly uninfected individual will test negative and was assessed in 86 unexposed European samples.

**Table 2:** Baseline characteristics of children enrolled into the study

Number of subjects	401
Mean age (months)	40.4 (± 19.2)*
Sex (male/female)	203/198
Mean temperature (°C)	38.4 (± 1.2)*
Mean haematocrit (%)	28 (± 3.1)*
Mean weight (Kg)	13.1 (± 3.2)*
% positive by microscopy	42.1% (169/401)
% positive by *stevor *PCR	71.1% (285/401)
% positive by *SSUrRNA *PCR	59.1% (237/401)
% positive by *msa-2 *PCR	50.1% (201/401)

* ± Standard deviation in parentheses

A total of 285 patients were detected to be infected with *P. falciparum *by the composite reference. Cohen's kappa coefficient showed that the *stevor *PCR has a complete agreement with the composite reference having a *Kappa *(95%CI) of 1.00 (1.000–1.000), followed by the *SSUrRNA *PCR κ = 0.718 (0.648–0.788) and the *msa-2 *PCR κ = 0.552 (0.478–0.627). The results of the detection of *P. falciparum *by the three PCR assays are shown in Table 3. All the 86 unexposed European samples tested negative with the three PCR assays thus, implying 100% specificity (Table 3). The specificity of microscopy could not be determined from the unexposed European samples because their slides were not available for microscopy.

**Table 3:** Sensitivity of the PCR-based assays for the detection of *P. falciparum*

	*stevor *PCR	*SSUrRNA *PCR	*msa-2 *PCR
Sensitivity	100%	83%	71%
Specificity	100%	100%	100%
Correctly classified	100%	87.2%	77.6%
Agreement (*Kappa*)^a, b^	1.00 (1.000–1.000)	0.718 (0.648–0.788)	0.552 (0.478–0.627)

^a^Composite reference compared with study assay; *Kappa *= 1 would indicate complete agreement.

^b^95% confidence intervals in parentheses.

To determine reproducibility, all samples that did not show identical result with the three PCR techniques were classified as discordant and were re-amplified. In all, 84 samples were re-examined and the PCR results obtained were 100% reproducible.

The diagnostic accuracy of the *stevor *PCR was significantly different to the other two PCR assays (*P *< 0.001) and, likewise, that of the *SSUrRNA *PCR was significantly different from the *msa-2 *PCR (*P *< 0.001).

## Discussion

Routine microscopic examination of Giemsa-stained blood smears is usually considered as the gold standard for malaria diagnosis. However, this technique requires highly skilled personnel and the information obtained by microscopy is limited when parasite levels are very low or when parasite morphology is altered. This has prompted the development of rapid diagnostic assays based on the detection of parasite antigens in whole blood and DNA based methods for the detection of parasites, mainly the clinically important *P. falciparum *infection. Rapid diagnostic tests, however, have low sensitivity at parasitaemia below 100 parasites/μl and have insufficient accuracy (Rubio, 2001).

PCR-based methods have been consistently shown to be powerful tool for malaria diagnosis [28-31]. Despite the fact that PCR-based assays have better sensitivity than conventional microscopy and antigen-based diagnostic tests [32], observations from this study show that their level of sensitivity could vary depending on the approach employed and the characteristic of the target sequence of the chosen assay. In this study, the *stevor *PCR was found to have the highest sensitivity for detecting *P. falciparum*, with 100% sensitivity and could correctly classify 100% of the population. This was followed by the *SSUrRNA *PCR with a sensitivity of 83% and could correctly classify 87% of the population, and then the *msa-2 *gene PCR with 71% sensitivity and could correctly classify 78% of the study population. Differences in sensitivity could thus, be correlated with the copy number of the amplified region although, sequence variations in target region of primers used might be a contributing factor. The *stevor*-based PCR is therefore, at a vantage position since it was designed to amplify fragments of a gene that has between 30 to 40 copies per haploid genome [33,34] compared to the *SSUrRNA *gene which has about four copies per haploid genome [35,36] or the *msa-2 *gene which is a single copy gene [37,38].

One limitation of this study though, is that it focuses on *P. falciparum *infections and does not address other species of human *Plasmodium*. However, *P. falciparum *demands particular attention because, it is the species of human malaria parasite that causes the most severe form of the disease and can kill with stunning speed.

## Conclusion

Despite having higher sensitivity and specificity in detecting *Plasmodium *infections, the use of PCR-based techniques in routine diagnosis is limited because of their logistical and technical difficulties [15]. They are labour-intensive and costly to maintain. However, PCR assays are invaluable tool in reference laboratories where quality assurance is required and, for public health surveillance in detecting submicroscopic asymptomatic infections. They could be particularly important in critical situations where a confirmatory assay with high sensitivity and specificity is needed for ruling out *P. falciparum *infection especially in non-immune travellers. The level of sensitivity of an assay though, could vary depending on the characteristics of the target sequence and the approach employed. This study shows that PCR assays with target to multiple copy sequences are more sensitive, the larger the copy number. As the scale of field studies grow, PCR diagnosis of malaria will play an increasing role in epidemiology with the development of high-throughput techniques that could facilitate large-scale analysis of samples within a short period.

## Authors' contributions

SIO contributed to the study design, data collection and analysis, interpretation of the results and preparation of the manuscript.

HOA contributed to the study design, data collection, interpretation of the results and preparation of the manuscript.

GCM contributed to the collection of data preparation of the draft manuscript.

EK contributed to the analysis of data and interpretation of the results.

PGK contributed to the study design, interpretation of the results and preparation of the manuscript.

JFK contributed to the study design, data analysis, interpretation of the results and preparation of the manuscript.

All authors read and approved the final manuscript.
